# Beyond hybrid professionals: evidence from the hospital sector

**DOI:** 10.1186/s12913-019-4442-1

**Published:** 2019-09-05

**Authors:** Marco Sartirana

**Affiliations:** 1 CERGAS - Centre for Research on Healthcare Management, SDA Bocconi School of Management, Bocconi University, Via Rontgen 1, 20136 Milan, Italy; 2 Utrecht School of Governance, Utrecht University, Utrecht, The Netherlands

**Keywords:** Reforms, Hospitals, Doctors, Professionalism, Hybrids, Italy

## Abstract

**Background:**

The involvement of doctors in managerial roles seems to be the solution to reducing the friction between traditional professionalism and modern organizational paradigms. However, these “hybrid” professionals responded in different ways to these conflicting demands, and we need to better understand the contextual factors that explain such variation.

**Methods:**

The paper studies hybrid professionals in a hospital characterized by numerous organizational changes. The site is located in Italy, a country in which healthcare organizations have been exposed to managerial reforms for years but where the degree to which professionals embraced management varies. A longitudinal case study was performed that involved gathering data through multiple sources of evidence to understand the complex organizational dynamics that take place in the hospital.

**Results:**

The analysis shows that the taking up of hybrid managerial roles is enabled by a number of interrelated features of the social/organizational context. Professionals willing to become hybrids were favored by the support provided by the organization. While for those doctors initially more reluctant towards medical management, distinctive contextual factors, in particular, the presence of space for interaction with colleagues within the professional domains but beyond disciplinary boundaries, was of key importance. This second group also proved capable of interiorizing organizational values and practices in a reconfigured way.

**Conclusions:**

In order to understand hybridization, it is necessary to look *beyond* hybrids at the context surrounding them. This study provides evidence for scholars and practitioners willing to understand how medical management is evolving and how this transition can be supported, and it contributes to the literature on hybrid managers by showing how contexts facilitating social interactions enable professionals’ hybridization.

**Trial registration:**

The article does not report the results of a health care intervention on human participants, and material used in the research did not need ethical approval according to Italian law.

## Background

### Introduction

A number of today’s healthcare challenges require medical professionals with the capacity to reconcile apparently contrasting values and priorities. The provision of the best quality of care and the use of the latest technologies for all patients faces the pressures of cost containment and efficiency needs. The increase of chronic illness and co-morbidities, calling for multidisciplinary and patient-centered settings, clashes with the traditional specialization of healthcare organizations, where service provision is organized according to rigid disciplinary boundaries. The spread of evidence-based medicine and accountability for outcomes calls for transparent measuring and reporting of results, in contrast with traditional performance management and people management systems based on informal relationship within the professional group [1–4].

To face these and other challenges, a number of health systems have fostered the development of “hybrids”, i.e., medical professionals engaged in managing professional work, colleagues, and other organizational resources [5–7]. They can be found at different levels, from C-level “physician executives” [6, 8] to frontline managerial roles. In particular, this paper focuses on clinical directors, i.e., medical management roles heading intermediate management structures and coordinating multiple medical specialties, with the aim of increasing clinical governance, control costs, and develop care integration and patient-centeredness [9]. As professionals called to manage colleagues and resources while at the same time retaining professional legitimacy within the group of peers (and, in many cases, their clinical practice), they have been studied as a paradigmatic example of hybrids in healthcare [10]. Pioneered at John Hopkins Hospital in the 1970s clinical directors have diffused among health systems globally, from the US to the UK, Canada, New Zealand and Australia, to other countries in Northern, Central and Southern Europe [11].

Hybrids have been seen as a way to reduce the friction between traditional professional values and new organizational paradigms. On the one hand, professionalism, described as based on the autonomy granted to professionals in order to apply their specialized knowledge to treat complex cases, while their behavior is collectively supervised and controlled within their professional group [12, 13]. On the other hand, management logics, which emphasize values like efficiency, cost-effectiveness, and accountability to patients and the public [14]. Research has shown that professionals taking up managerial roles responded to these conflicting demands in different ways. While some of them formally took on management roles without substantially performing management practices [15–17], many others fully enacted their hybrid roles as they succeeded in finding meaningful combinations of professional and managerial values [18]. However, the antecedents of developing hybrid roles require further research [8] and scholars have been called to look beyond hybrids’ response strategies and study “the agency and social interaction processes that shape these responses” [19: 285].

Therefore, this study aims to achieve a better understanding of the conditions and contextual factors at the organizational level that impact hybridization, i.e., the process through which professionals take up hybrid roles-identities. This is particularly relevant in the hospital sector context, which is undergoing a number of rapid changes that are questioning the traditional organization of professional work [3], and in which “our understanding of the nature and impact of reforms and how they are re-shaping the relationship between medicine and management remains limited” [4]. This is a relevant topic not only in theory but also in practice, as healthcare managers and policymakers, who are struggling with the engagement of professionals in management, are keen to understand the reconfigurations of medical management and how to create supportive organizational environments for hybrids [20].

Because I wished to analyze medical management in a country which has “imported” New Public Management reform templates from abroad, the paper studies hybrid roles outside the Anglo-Saxon and the Scandinavian world. In particular, clinical director roles - which were part of a global trend that introduced, via a “translation” process, a standardized medical management model to most Western health systems [21] - encountered political and cultural resistance in a number of European countries [11]. As a consequence, there is evidence that despite management practices that are now embedded in healthcare organizations, the degree to which professionals have embraced new hybrid roles and identities is still limited, and different European health systems have shown mixed evidence with reference to the responses to hybrid management innovation [22, 23]. For instance in France it has been found that doctors heading the newly introduced “medical poles” struggle to achieve legitimacy by leveraging pre-existing professionals networks [24], while in Germany the new hybrid model is confronted by existing (sedimented) practices, as senior doctors feel their ascendancy threatened [25].

In order to explain this variability, previous research has called for a contextualized understanding of environmental and organizational antecedents, as well as individual factors, in explaining doctors’ capacity to take on managerial roles [26]. This qualitative longitudinal study in an evolving hospital environment characterized by different managerial interventions over time allows for an in-depth understanding of the relationship between hybrids and their complex organizational context. The site is a large tertiary hospital which underwent healthcare sector reforms in the 1990s. It introduced performance management systems, favored the involvement of doctors on the hospital board, and set up intermediate organizational layers (clinical directorates) headed by professionals responsible for the use of resources. More recently, the hospital witnessed the introduction of patient-centered logics and moved to a new building with a redesigned layout.

This analysis bridges health services research and health policy with organizational studies, responding to the calls to develop research with a stronger engagement between the three approaches [4, 27]. The paper is organized as follows: after presenting the theoretical background of the study, the research context and the methodology, results are illustrated and discussed, and implications for healthcare policy and management are drawn.

### The evolution of healthcare professionalism and the missing role of context

The relationship between management and professionalism in healthcare is a rich and dynamic field of study, following the continuous evolution of professional practices and professional organizations worldwide. Initially, research anchored in the sociological tradition pictured managerial reforms as a process in which professional culture was subjugated by managerial hegemony; alternatively, it argued that professionals were capable of resisting managerial dominance by open or subtle opposition strategies [7, 28, 29]. Over time, research has progressively overcome this hegemony-resistance framework shifting instead to the study of hybrid professionalism and hybrid professionals, and the strategies used to juxtapose and eventually blend the different values, logics, and practices [14]. Hybrids, and especially clinical directors, who usually work as part-time practitioners and part time managers of their colleagues, in the position of a primus inter pares, were found capable of mediating managerial and clinical demands. They have been asked to contribute to hospital-wide strategy making, management of resources and coordination of care linking professional practice with organizational objectives. This capacity was described as the mediation or blending of alternative logics [30], or the creation of “two-way window” roles [31]. Accordingly, hybrid professionalism has been described as the co-product of clinical and organizational values [32]. However, staying in hybrid roles is not easy and individual responses as well as professionals’ level of engagement vary, and in multiple cases doctors passively engaged in management, or coopted management instrumentally [7].

The variety of individual responses was due to a significant extent to individual determinants, in particular personal willingness and motivation toward management, as well as ability and capacity. For instance, McGivern et al. [10], in line with other scholars from different Western countries [15, 16], distinguish between willing hybrids, doctors who are enthusiastic about and take an entrepreneurial approach toward the managerial role, willing to redefine their professional identity and challenge traditional professional practices; and incidental or reluctant hybrids, professionals who maintain a more passive and reactive attitude, looking at management as something which “needs to be done”. Recently, scholarship has suggested going one step further, observing that some medical managers’ activities are increasingly both managerial *and* professional. The reorganization of clinical practice or the planned efforts to increase service quality are activities that are developed in order to respond to professional rather than to managerial values, acknowledging the evolution of patients’ needs, and societal and cultural changes. While the notion of hybridity reflects the juxtaposition and combination of different values, there has been a move *beyond* hybridity [14], in which organizing becomes an intricate part of professional work and managerial and professional discourses overlap [33, 34].

Yet, although this rich literature thoroughly covers the different responses of professionals to management and the influence of individual values, preferences, and motivations, our understanding of the contextual conditions explaining such variation remains limited [4]. Moreover, while existing studies hint at antecedents of effective hybridization [10], they provide little information regarding this. In particular, while Noordegraaf [18] suggested exploring the relational dimension of professionalism and its links with the outside world, organizational rationales, and other professions, we know relatively little with reference to the role of the social/organizational context on these individual dynamics. As argued by Denis et al. [19], there is a need to look beyond hybrids’ responses and rather study how these are shaped by agency and social interactions.

Studies in the medical field tend to look only at interactions taking place within homogeneous professional communities, as in the case of Pratt [35], who has analyzed the importance of socialization and role modelling in the development of young doctors’ identities. Other authors have studied hybrids and the construction of their role among colleagues and their legitimization as “primus inter pares” [31, 36]. In contrast to this literature, the recent work by Reay et al. [37] demonstrates the potential for change to be orchestrated by *others*, i.e., business managers, who supported doctors in incorporating managerial values. This work is extremely interesting, but it studies general practitioners, who are professionals without major managerial responsibilities and work outside complex organized settings. This paper aims to fill this gap by giving empirical attention to the dynamics of medical managers’ effective role-taking and the influence of the organized context in which they take place, in order to understand how these transitions can be supported.

As a consequence, the research question of this study can be synthesized as “*how do medical managers hybridize over time, and if and how this evolution is influenced by the social/organizational context in which professionals work*?”. I do this by studying hybrids in an evolving hospital organization, as modern hospitals are suitable places to examine the impact of context on medical professionalism. They are complex and institutionalized environments in which professional work and roles are affected by managerial reforms [21] and which continuously expose professionals to new types of interactions with colleagues and non-medical managers.

## Methods

I conducted this case study [38, 39] with the goal of developing our understanding of hybrid professionalism in organized contexts. This in-depth longitudinal analysis, allowed by qualitative methodologies and multiple sources of evidence, was meant to grasp the complex organizational dynamics taking place in a hospital setting. Similar research approaches were adopted by a number of previous studies on the topic [e.g., 17, 36], and collecting data over time allowed to take into consideration the impact of the changes to the work context determined by the hospital’s relocation.

The hospital I studied is a critical case to analyse hybrid doctors in management as it has been exposed to management practices for the last 25 years. In recent years, it has undergone a relevant development and empowerment of clinical director roles as well as quality management initiatives and clinical pathways. Furthermore, while conducting the study, the hospital relocated from the old pavilion-based physical structure to a newly built site, designed to favour inter-unit collaboration and care integration. The following is a description of the history of medical management in the Italian NHS and the managerialization at the hospital before 2012 when the study began, and a synthesis of the main organizational changes that took place during the study.

### Medical management in the Italian hospital sector

The Italian hospital sector has a long history of medical management. Unit chiefs have traditionally been accountable for unit level managerial responsibilities, and doctors working in hygiene departments have offered executives support for a number of hospital management and organizational issues [26]. With an aim to increase accountability for results and to favour clinical governance and collaboration within directorates, the healthcare sector reforms of the 1990s made the clinical directorate model compulsory in public hospitals of all regional health systems. Clinical directors (CDs) can now be found in every organization, although with varying degrees of engagement in the role. In some cases, the implementation process was poor and doctors took on the role formally rather than substantially [22]. Increasingly, in recent years, in addition to the new organizational structure, process management logics aimed at favouring the sharing of resources and transversal collaborations beyond the directorate boundaries have been present in healthcare policy guidelines [40]. Clinical pathways spread to all hospitals for the care of major diseases, and new organizational forms characterized by multidisciplinary departments and wards grouped according to patient care needs are now common [41]. However, the impact of these reforms has been questioned: while some Italian professionals appear enthusiastic about the new patient-centered approaches and its focus on quality, others blame the reforms for looser systems of responsibility, de-specialization, and further impoverishment of the medical status [23].

### Managerialization reforms at the hospital

The hospital is located in Northern Italy and with almost 1000 beds, 4000 employees, and a yearly budget of over 400 million euros, it is one of the largest in the area. It is in one of the Regions that, in recent years, has significantly invested in the managerialization of the healthcare system. For many years after the introduction of clinical directorates, dating back to the late 1990s, every unit director preserved “his own kingdom”. Specialty unit directors maintained a pivotal role in the organization and governed according to professional, rather than managerial, logics, and this was strengthened by a traditional pavilion architecture dating back to the 1930s, where wards, outpatient services, and operating rooms were fragmented. In this scenario, CDs’ managerial activity largely consisted of cost management. They were rarely active in strategy making or in the promotion of collaboration initiatives within the directorate or across directorates, proving unable to overcome conflict of interests or think beyond the boundaries of their specialty. A minority of CDs were willing to take on more managerial responsibilities but encountered the resistance of specialty unit directors, who were defensive and did not want to be governed by a peer. Furthermore, they faced opposition from hospital executives, who were prudent in delegating responsibilities and involvement in strategy making; for instance, the hospital board (a council of all CDs together with the top management team) was summoned only four times a year and never became a decision-making body. Also, the central unit of doctors specialized in hospital organization (the so-called “hygienists”) took care of operations management responsibilities, further reducing the need for professionals’ involvement in hybrid roles.

However, in recent years a number of changes has taken place. Firstly, with the support and supervision of non-medical managers, clinical pathways were introduced not only in transplant services - which had historically required inter-unit coordination - but also in cancer care, trauma care and a number of other major diseases. Furthermore, after the arrival of a new top management team in 2011, CDs were strongly empowered and transformed into an effective organizational structure. They were made accountable for the financial performance of the directorate and became increasingly involved in strategy making; the hospital board started meeting on a monthly basis, and executives began fostering CDs’ active participation in the decision-making process. For example, they were involved in planning the future hospital relocation, actively consulted on the design of the organizational structure, and made decisions concerning technological investments, the physical layout, and the relocation schedule. Meetings with the group of unit chiefs of the directorate increasingly dealt with clinical integration initiatives and other organizational issues. Accordingly, opportunities for effective socialization among clinical directors significantly increased. Finally, in 2013, the hospital was relocated from the historical pavilion structure to a newly-built site designed according to modern architectural concepts, including the integration of facilities and the optimization of logistics and patient flow according to process management logics. Medical offices for different units were placed side by side, with common meeting rooms. Operating theaters and outpatient services were concentrated and shared among different departments, and different clinical specialties started sharing the same nursing staff and, in some cases, the same inpatient beds.

### Data collection, analysis, and interpretation

I obtained access to the site thanks to a research partnership established between the hospital and the university. I gathered data over time from three different types of sources (Table 1): 1) semi-structured interviews with hospital senior medical and top managers; 2) observation at board meetings; 3) archival data. I had the opportunity to interview, at least once, all hospital CDs,^1^ doctors who had part-time involvement in management while maintaining clinical activity and the direction of a specialty unit. Therefore, interviewees were able to report on their experience of bridging medicine and management at different levels, ranging from clinical practice, to specialty unit management and clinical directorate management. I also interviewed the hospital’s top management team, including the CEO, the administrative and medical director, the chief nurse, as well as the chief hygienist and the three hygienists on his team, as the professionals that are most exposed to managerial interactions with CDs. Two CDs retired during the study period and were replaced while all other interviewees maintained their roles. Nine interviews, with the chief hygienist, the two newly appointed CDs and a share of CDs (selected with a purposeful sampling based on the relevance of data collected in the first round of interviews) took place after the relocation In total, 30 interviews were conducted from May 2012 to July 2014.

**Table 1:** Data sources by period

	*2012*	*2014*	Total
*Interviews with top management and chief nurse*	4		4
*Interviews with clinical directors*	13	8	21
*Interviews with hygienists*	4	1	5
*Observation at board/executive meetings*	14 h	4 h	18 h
*Analysis of archival data (resumes, organizational charts and strategic plans)*	Yes	Yes	

The research approach allowed for a plurality of perspectives and in-depth insights into the way doctors struggled to bridge medicine and management over time. A constant comparison technique was employed, repeatedly comparing data across informants and over time. Anonymity was guaranteed. All interviews were held in Italian, with the average length of one hour. The interview protocol was based on existing literature on medical managers (see in particular [42]). The topic list for interviews with CDs, fine-tuned after a pilot interview, included open-ended questions on the contents of their managerial activities, the hybrid role, and the contextual influences on entering the role and if/how this changed over time. Other respondents were asked to report on their experience with CDs’ roles, the nature of the interactions with them, and the evolution of both. Interviews were conducted on site by the author, digitally recorded and summarized in order to condense the most relevant findings emerging from respondents [43].

Interview data were triangulated with the analysis of old and new organizational charts, strategic plans and the résumés of interviewees, as well as observation at hospital strategic meetings. The researcher observed four three-hour board meetings in 2012, with the aim of gaining a deeper knowledge of the hospital executives’ management styles, the nature of the contribution of different CDs to discussions, and explicit and non-verbal reactions to managerial decisions. Further evidence was collected by the author who, in 2014, participated in two two-hour meetings about the development of care pathways with top management and a selection of CDs.

The qualitative data analysis was developed by identifying relevant concepts in the empirical material, grouping them into categories emerging from the words and ideas expressed by informants, and using the insiders’ point of views as the starting point of the analysis. Statements of findings were made when corroborated across multiple informants or data sources. The interpretation was then developed in an iterative process commuting between existing organizational theory on hybrids in healthcare and data. In line with this, I was guided by the literature that identified doctors’ alternative responses to management, with a distinction between willing and incidental medical hybrids [10, 15, 16], also reported in a previous work [42]. With the understanding that evidence presented variability across a continuum rather than a clear-cut dichotomy, in the results section data is presented according to this distinction. At the same time, I induced my understanding of the influence of organizational context on hybrids’ role-taking from the empirical analysis.

## Results

All CDs at the hospital were highly influential clinicians, with the professional legitimacy to occupy their positions. The analysis of resumes showed that they had been unit chiefs for years, and had served as presidents of scientific societies or members of editorial boards of international journals. Furthermore, all of them had received a general management training program, legally prescribed for all specialty unit directors, of about 15 days. However, while talking with them about their hybrid role and the nature of their professional-managerial commitment, as well as while gathering the views of top managers and hygienists, a small group of CDs emerged (about one third of the CDs interviewed) who saw their role as providing an opportunity to initiate change, set a direction, and contribute to the strategy of the hospital. In line with previous research, it was evident that these professionals were willing to perform as hybrids, while a second larger group of CDs appeared more reluctant towards the managerial engagement. However, the role played by the organizational context in the development of these two groups of hybrids was significantly different.

### Willing hybrids and the support from executives

Professionals in the first group focused on setting a direction for the directorate and coordinating the activity of other specialties as primus inter pares. When their interactions were observed at board meetings, they showed confidence in using managerial tools, in talking about directorate planning, and contributing to strategy making, as shown by a discussion developed by one of them during a board meeting regarding the opportunities to attract patients from abroad to increase private practice. They were comfortable in their roles, finding a meaningful professional mission in the new position, and did not experience relevant dilemmas between professional and managerial values, as underlined by the following quotes:

“I think I started to behave like a clinical director the day after my graduation, that’s why I do not find any difference … it is just a responsibility at a higher level - more nurses, more doctors, more organization, more people coming to me with problems.” (CD16)

“My mission is to motivate people to achieve difficult goals, to have people that come to work every day and think, ‘what can I do for patients *and* the hospital?’”. (CD9)

All of them reported that in order to fully enter their role, the relationship with top managers and the support provided by them was of key importance. In the past, they had had few interactions with executives, had been scarcely involved in hospital decision making and they had been frequently bypassed by top managers, which undermined their legitimacy in front of peers:

“In the past unit chiefs knocked directly on the CEO door to be listened to” (CD8)

However, they greatly appreciated the active responsibilities attributed to CDs by the new top management team and the fact that board meetings had become an opportunity to share problems and communicate with the CEO, as this represented the chance to fully enter their position as CDs. They started receiving backing from executives in making complex decisions, and real delegation of responsibilities, which allowed them to fully enter their roles.

“At the beginning, the board was only a place to get to know each other, and to discuss problems of clinical quality, now it is different … we are responsible for administrative and financial matters, and this has greatly improved our relationship with the top managers.” (CD4)

“In the past the council of directors was useless. Executives pretended to involve us. Now they really want to do it; they want to listen to our opinions.” (CD8)

“I call for a vision … and many of them already are capable of thinking strategically … It is very important to support them … e.g. if someone asks to be trained in some managerial areas, like time management; and they are not alone , the medical director is beside them.” (Top Manager 1)

All of them acknowledged the importance of the new forms of interaction with colleagues favored by the introduction of clinical pathways or by the new layout. However, none of them reported these elements as major factors in explaining the evolution of their medical role. As a matter of fact, in many instances, they had pioneered multidisciplinary projects years before; therefore they perceived that these features had always been part of their professional practice:

“Medical education, quality management, patient-centeredness initiatives … these are things that have always been dealt with in our directorate.” (CD14)

### Incidental hybrids and spaces for professional interaction

Most doctors in this second group preferred clinical practice to management activities; they felt uneasy in dealing with strategy making and in contributing to complex decision making. For instance, during board meetings they mostly referred to issues related to budget cuts in a reactive way, but without suggesting more strategic oriented proposals:

“For some of them board meetings are just the place for complaints.” (Hygienist 1)

They expressed a more cautious attitude, viewing their role as keeping good relations with other unit chiefs and merely channeling information. And despite the backing of the top management, their attitude had not changed over time, as they appeared mostly bound to their original specialty, and maintained a professional mindset with a limited capacity to take on major responsibilities in front of colleagues.

“At the beginning, it is new and unknown, it is a challenge … but then you realize that it interferes with your clinical practice … it is a difficult choice for those who have clinical activity, and sometimes I think that I’d have more fun doing other things.” (CD5)

“I spend most of my time in clinical work because that is what I like. I was afraid that they would have asked me to leave the clinical practice, and in that case, I would have resigned … it is that management clashes with my mentality a bit.” (CD10)

“[Some CDs] have problems in managing resources; some are not capable, some are afraid of conflicts, others are purists and do not want to get dirty with money because they say it is not their role.” (Top Manager 2)

“Some CDs perform the role just to survive … they can lack the commitment, or the competences.” (Top Manager 1)

However, these professionals also experienced some kinds of change in the way they organized service provisions, and they referred to the adoption of practices like collaboration, resource pooling, programming, standardization of work, and stakeholder management. For instance, a CD in a directorate meeting reported with competence and satisfaction the initiatives he had put in place to favor communication and integration between hospital and primary care professionals in the provision of cancer care. As a matter of fact, these very professionals that were unwilling to engage in the strategy making and financial responsibilities associated with the role, were indeed performing managerial work practices. And these practices were referred to as a having an intrinsic professional – and not only organizational - nature:

“There are management activities that are part of the clinical work because they are about reorganizing and rationalizing clinical activity.” (CD11)

“If I have bad relationships with a City Council, that means not only that I have an economic problem, but that the patients who live in that area will be treated poorly. [ … ] Building visibility and establishing pathways in collaboration with society is something necessary today … it is part of clinical work.” (CD13)

These doctors commented on how this change was favored by a number of factors, which included the importance of working together within clinical pathways, initially developed by the hospital in transplants and then extended to in the care of other illnesses, which resulted in work cross-boundary work and in an interdisciplinary way in providing clinical care. Furthermore, they reported the importance of interaction with other organizational professionals, such as the hygienists, the quality office, or the pharmacists.

“Now I have quality people within the directorate … and our activities are now linked with the quality system. Before when a clinical director made a decision... it was rarely translated into practice, now it is different”. (CD2)

The new physical layout had also progressively, sometimes coercively, redefined their way of working by favoring frequent interactions with colleagues. The following quotes, from the second round of interviews, show the impact of the new physical layout on hybrids’ behaviors:

“Now it is easier not to be schizophrenic, I mean divided between the part [i.e., the specialty] and the whole, and rather think in a unitary way … . For instance, having a multidisciplinary organization in trauma care, as well as the new spaces, facilitate a unitary vision, not bound to the specialty.” (CD5)

“Before, everyone was in a different pavilion and had his own kingdom. Now we have common spaces, shared operating rooms … and we can dialogue on a daily basis.” (CD7)

Of course, some variation was found across areas of the hospital. For instance, surgical CDs proved to be more resistant to the reshaping of work practices than other professionals. As shown in the following quote, although some new work processes and shared standards were introduced, the traditional autonomy and self-regulation proved hard to dismantle.

“For us surgeons, it is more difficult … in operating rooms change takes more time … going from everyone having their own treasure to a situation where the treasure is common and shared on the basis of need is more difficult.” (CD5)

“They are afraid of losing power, because they cannot say ‘this ward is my own territory’ any more” (Hygienist 1)

## Discussion

The evidence that was collected interviewing CDs and managers surrounding them, as well as from observation, highlights the taking up of hybrid managerial roles as enabled by a number of interrelated features of the social/organizational context. Therefore, in order to fully understand hybridization, we should not only acknowledge the role of professionals’ values, traits, and motivation but also look *beyond* hybrids at the context surrounding them. In particular, the analysis found that different groups of hybrids responded differently to the interactions taking place in the organizational context.

On one side, the engagement of “willing” hybrids in their role was made possible not only by individual features but also thanks to the support they received in the organization, in line with what was found by the recent work by Reay et al. [37]. Triangulating the perceptions of CDs, hygienists and top managers, I found that interactions of CDs with executives, involvement in decision making and backing of difficult decisions, were the features of a context providing the opportunity for managerial action, which was an essential condition for the full engagement over time of these professionals in their hybrid roles.

On the other side, “incidental” hybrids were mostly bound to the traditional professional mindset and values, they were not motivated or capable enough to fully take up the decision making responsibilities over finance and hospital-wide planning, even with the support they received within the organization. However, doctors in this second group showed the capacity to effectively engage - over time - in other forms of management and organizational commitments. Rather than activities like strategy making or monitoring, these professionals engaged in quality initiatives, such as service reorganization, staff empowerment or accountability to patients. For these doctors, a different factor played a key role, i.e. a social and organizational context providing *space for interaction* with other professionals from different specialties. This included: occasions to come in contact with other organizational professionals like pharmacists, hygienists or quality staff; the involvement in pathways and multidisciplinary forms of organizing; the collaboration among disciplines required as a consequence of the pooling of resources and the redefinition of working spaces. Therefore, changes of professional identities are driven not only by interaction with colleagues within the professional group [35] or with business managers [37] but by the interaction with professionals beyond the disciplinary boundaries.

What I found with reference to this second group develops what Noordegraaf [14] hints at when he argues that “redefined” and “post-hybrid” medical managers are not primarily collaborating with executives but rather establishing connections with other colleagues, patients, and external stakeholders within their professional domains. As shown in the study, reconfigured forms of professionalism incorporating values like collaboration, quality or accountability to patients appear more easily accessible to doctors, and not only to an elite group of professionals capable of getting and willing to get involved in strategy making and performance management. However, in order to favor this evolution, “romanticized appeals to professionalism” are not enough, as argued by Martin et al. [44]. Instead, professionals need to be surrounded by contexts purposefully designed by managers to support interaction, and in which change in work processes is enabled or - in some cases - even coerced. Further examination of the precise mechanisms through which these organizational and social elements function represents a fruitful avenue for future research.

This direction of change towards a reconfigured form of medical management looks promising for all those hospitals in Western countries that have been struggling with managerial reforms and exposure to organizational reconfigurations that aim to increase care integration and patient centeredness. This is why these findings from the Italian experience can provide theoretical insights to other Southern and Central European countries experiencing professional resistance to the introduction of managerial values and structures in hospitals [45].

Indeed, variation emerged as some clinical directors showed resistant behaviors. This proves that the evolution of professionalism is not a straightforward process, as some forms of medical professionalism offer fewer opportunities for embedding organizational values and practices. As shown in the case of surgeons (see also Correia and Denis [17]), combinations of professional and managerial principles may remain uneasy. And progress to a reconfigured professionalism is not straightforward but requires time and long-lasting efforts.

Of course, the study has limitations linked to the nature of the methods used. Although the case study proved effective in offering a in-depth understanding of the hospital’s organizational context, the analysis has an explorative nature and uses information from a single organization. Thus, future research could benefit from multiple case studies. Furthermore, comparative international evidence would shed more light on the effects on medical management models in other countries. In addition, the examination of the effectiveness of the different forms of hybrid medical managers on organizational or clinical outcomes represents a necessary path for future research that could guide policymaking and management. Finally, although all doctors I interviewed had been involved in formal management training paths, these were not referred to as major triggers for managerial engagement. Therefore, the effectiveness of healthcare management courses and how it can be increased thanks to supportive organizational environments might be fruitfully explored.

## Conclusions

By focusing on a hospital case study in the Italian NHS, this paper has illustrated that the development of hybrids is enabled by different forms of interaction with multiple organizational actors. In particular, I have shown how incidental hybrids, if properly supported, can take up organizational principles, which are not seen by doctors as “alien” with respect to professional practice, but rather as both professional *and* organizational.

In terms of implication for healthcare top managers, it is clear that a careful selection of candidates for hybrid positions is of paramount importance, and whenever possible, it is vital to find management-oriented professionals. However, I showed that willing hybrids should be provided with opportunities for interactions with top managers, effective involvement, and backing in complex decision making. Likewise, incidental hybrids, if provided with “spaces” for interaction with other professionals from different specialties (e.g. shared wards, care pathways or contacts with pharmacists, hygienists, or quality staff) can effectively engage in service reorganization, quality management and patient centered redesign initiatives.

Understanding such dynamics is relevant not only for scholars and executives but also for healthcare policymakers interested in the outcomes and implementation challenges of reforms in a number of European countries. This was the reason behind the intent to combine the different but complementary approaches of health services research and organization studies, capable of providing a richer view of what medical management actually is.

## Data Availability

The dataset used during the current study are available from the corresponding author on reasonable request.

## Abbreviations

CD: Clinical Director
CEO: Chief Executive Officer
NHS: National Health System

## Footnotes

1 When the study was conducted, the organizational chart identified 11 ‘managerial’ clinical directorates and 5 ‘support’ clinical directorates. Each ‘managerial’ directorate included an average of five specialty units, ranging from a minimum of two to a maximum of ten. The CD of the preventive medicine directorate was excluded as, due to the different type of clinical activity, it was considered not relevant for the analysis. In 2013, during the study period, a minor reorganization took place which made minor changes in the ascription of units to directorates.

## References

[1] Dwyer AJ (2010). Medical managers in contemporary healthcare organisations: a consideration of the literature. Aust Health Rev.

[2] Spehar I, Frich JC, Kjekshus LE (2012). Clinicians’ experiences of becoming a clinical manager: a qualitative study. BMC Health Serv Res.

[3] Lega F, Calciolari S (2012). Coevolution of hospitals and patients: how changing epidemiology and technology advances drive organisational innovations and lay new challenges. J Healthc Manag.

[4] Kirkpatrick I, Kuhlmann E, Hartley K, Dent M, Lega F (2016). Medicine and management in European hospitals: a comparative overview. BMC Health Serv Res.

[5] Fitzgerald L, Dufour Y (1998). Clinical management as boundary management, a comparative analysis of Canadian and U.K. healthcare institutions. J Manag Med.

[6] Montgomery K, Physician executives. The evolution and impact of a hybrid profession. In: Blair J, Fottler M, Savage G, editors. Advances in Health Care Management Volume 2 (215–241): Emerald Group Publishing Limited; 2001. http://www.emeraldgrouppublishing.com/products/books/series.htm?id=1474-8231.

[7] Numerato D, Salvatore D, Fattore G (2011). The impact of management on medical professionalism: a review. Sociol Health Illn.

[8] Hoff TJ (2001). Exploring dual commitment among physician executives in managed care. J Healthc Manag.

[9] Braithwaite JM, Westbrook T, Iedema R, Mallock NA, Forsyth R, Zhang K (2005). A tale of two hospitals: assessing cultural landscapes and compositions. Soc Sci Med.

[10] McGivern G, Currie G, Ferlie E, Fitzgerald L, Waring J (2015). Hybrid manager-professionals’ identity work, the maintenance and hybridization of professionalism in managerial contexts. Public Adm.

[11] Neogy I, Kirkpatrick I (2002). Medicine in management: lessons across Europe.

[12] Abbott A (1988). The system of professions: an essay on the division of expert labor.

[13] Freidson E (2001). Professionalism: the third logic.

[14] Noordegraaf M (2015). Hybrid professionalism and beyond: (new) forms of public professionalism in changing organizational and societal contexts. J Prof Org.

[15] Forbes T, Hallier J, Kelly L (2005). Doctors as managers: investors and reluctants in a dual role. Health Serv Manag Res.

[16] Von Knorring M, de Rijk A, Alexanderson K (2010). Managers' perceptions of the manager role in relation to physicians: a qualitative interview study of the top managers in Swedish healthcare. BMC Health Serv Res.

[17] Correia T, Denis JL (2016). Hybrid management, organizational configuration, and medical professionalism: evidence from the establishment of a clinical directorate in Portugal. BMC Health Serv Res.

[18] Noordegraaf M (2007). From “pure” to “hybrid” professionalism. Present-day professionalism in ambiguous public domains. Adm Soc.

[19] Denis JL, Ferlie E, Van Gestel N (2015). Understanding hybridity in public organizations. Public Adm.

[20] Kuhlmann E, Rangnitt Y, von Knorring M (2016). Medicine and management: looking inside the box of changing hospital governance. BMC Health Serv Res.

[21] Kirkpatrick I, Bullinger B, Lega F, Dent M (2013). The translation of hospital management reforms in European health systems: a framework for comparison. Br J Manag.

[22] Lega F (2008). The rise and fall (acy) of clinical directorates in Italy. Health Policy.

[23] Liberati EG, Gorli M, Scaratti G (2015). Reorganising hospitals to implement a patient-centered model of care: effects on clinical practice and professional relationships in the Italian NHS. J Health Organ Manag.

[24] Vinot D (2014). Transforming hospital management à la francaise: the new role of clinical managers in French public hospitals. Int J Public Sect Manag.

[25] Bode I, Maerker M (2014). Management in medicine or medics in management? The changing role of doctors in German hospitals. Int J Public Sect Manag.

[26] Sartirana M, Prenestini A, Lega F (2014). Medical management: hostage to its own history? The case of Italian clinical directors. Int J Public Sect Manag.

[27] Currie G, Dingwall R, Kitchener M, Waring J (2012). Let’s dance: organization studies, medical sociology and health policy. Soc Sci Med.

[28] Kitchener M (2002). Mobilizing the logic of managerialism in professional fields: the case of academic health Centre mergers. Organ Stud.

[29] Waring J, Currie G (2009). Managing expert knowledge: organizational challenges and occupational futures for the UK medical profession. Organ Stud.

[30] Pache AC, Santos F (2013). Inside the hybrid organization: selective coupling as a response to competing institutional logics. Acad Manag J.

[31] Llewellyn S (2001). Two way windows: clinicians as medical managers. Organ Stud.

[32] Schott C, Van Kleef DD, Noordegraaf M (2016). Confused professionals? Capacities to cope with pressures on professional work. Public Manag Rev.

[33] Kirkpatrick I, Noordegraaf M, Empson L, Muzio D, Broschak J, Hinings B (2015). Hybrid professionalism: the re-shaping of occupational and organisational logics. The Oxford handbook on professional service firms.

[34] Olakivi A (2017). Niska M rethinking managerialism in professional work: from competing logics to overlapping discourses. J Prof Org.

[35] Pratt MG, Rockmann KW, Kaufmann JB (2006). Constructing professional identity: the role of work and identity learning cycles in the customization of identity among medical residents. Acad Manag J.

[36] Witman YG, Smid AC, Meurs PL, Willems DL (2011). Doctor in the lead: balancing between two worlds. Organization.

[37] Reay T, Goodrick E, Waldorff S, Casebeer A (2007). Getting leopards to change their spots: co-creating a new professional role identity. Acad Manag J.

[38] Eisenhardt KM (1989). Building theories from case study research. Acad Manag Rev.

[39] Yin RK (2009). Case study research: design and methods.

[40] Lega F, Sartirana M (2016). Making doctors manage … but how? Recent developments in the Italian NHS. BMC Health Serv Res.

[41] Tozzi VD, Longo F, Pacileo G, Salvatore D, Pinelli N, Morando V (2014). PDTA standard per le patologie croniche.

[42] Sartirana M. Opportunity does matter: supporting public professionals in management. In: Pedersen A, Waldorff S, Ferlie E, Fitzgerald L, editors. Managing change: from health policy to practice. London: Palgrave; 2015.

[43] Weiss RS (1994). Learning from strangers: the art and method of qualitative interview studies.

[44] Martin GP, Armstrong N, Aveling EL, Herbert G, Dixon-Woods M (2015). Professionalism redundant, reshaped, or reinvigorated? Realizing the "third logic" in contemporary health care. J Health Soc Behav.

[45] Kuhlmann E, Burau V, Correia T, Lewandowski R, Lionis C, Noordegraaf M, Repullo J (2013). A manager in the minds of doctors: a comparison of new modes of control in European hospitals. BMC Health Serv Res.

